# Biocontrol of Fungal Pathogens of Winter Oilseed Rape (*Brassica napus* L.) Using Plant Extracts (*Allium sativum*, *Helianthus tuberosus*) and Bacterial Fermentation Supernatants (*Paenibacillus*, *Enterobacter*)

**DOI:** 10.3390/molecules31183195

**Published:** 2026-09-10

**Authors:** Jakub Danielewicz, Ewa Jajor, Joanna Horoszkiewicz, Marek Korbas, Ilona Świerczyńska, Łukasz Siekaniec, Marcin Podleśny, Marzena Mikos-Szymańska, Marta Klimczyk, Tomasz Szymczak, Jagoda Kucharska, Monika Kobiałka, Jan Bocianowski

**Affiliations:** 1 Department of Mycology, Institute of Plant Protection, Władysława Węgorka 20, 60-318 Poznań, Poland; e.jajor@iorpib.poznan.pl (E.J.); j.horoszkiewicz@iorpib.poznan.pl (J.H.); m.korbas@iorpib.poznan.pl (M.K.); i.swierczynska@iorpib.poznan.pl (I.Ś.); 2 Regional Experiment Station in Rzeszów, Institute of Plant Protection, Boya-Żeleńskiego 15, 35-105 Rzeszów, Poland; l.siekaniec@iorpib.poznan.pl; 3 Fermentation Technology Department, Institute of Agricultural and Food Biotechnology, Rakowiecka 36, 02-532 Warszawa, Poland; 4 Grupa Azoty Zakłady Azotowe “Puławy” S.A., Al. Tysiąclecia Państwa Polskiego 13, 24-110 Puławy, Poland; marzena.askamik@wp.pl (M.M.-S.); klimczyk.marta89@gmail.com (M.K.); tomasz.szymczak@grupaazoty.com (T.S.); jagoda.kucharska@grupaazoty.com (J.K.); monika.kobialka2@grupaazoty.com (M.K.); 5 Department of Mathematical and Statistical Methods, Poznań University of Life Sciences, Wojska Polskiego 28, 60-637 Poznań, Poland; jan.bocianowski@up.poznan.pl

**Keywords:** *Alternaria* spp., biological control, biostimulation, *Botrytis cinerea*, *Brassica napus*, *Enterobacter*, garlic, Jerusalem artichoke, *Paenibacillus*, *Phoma lingam*, *Sclerotinia sclerotiorum*

## Abstract

Winter oilseed rape (*Brassica napus* L.) production is constrained by a complex of fungal pathogens, including *Alternaria alternata*, *A. brassicicola*, *Botrytis cinerea*, *Phoma lingam* and *Sclerotinia sclerotiorum*, which are conventionally managed with synthetic fungicides such as prothioconazole. This study evaluated the biocontrol and biostimulant potential of two plant extracts (garlic, *Allium sativum*; Jerusalem artichoke, *Helianthus tuberosus*) and two bacterial fermentation supernatants (*Enterobacter* sp. and *Paenibacillus* sp.) against these pathogens under in vitro, greenhouse and field conditions (2022–2024, two locations: Winna Góra and Rzeszów, Poland). In vitro, the *Paenibacillus* sp. supernatant showed the broadest and strongest activity, with inhibition of mycelial growth comparable to prothioconazole for several pathogens, particularly *A. brassicicola* and *B. cinerea*. Garlic extract was most effective against *S. sclerotiorum* and *B. cinerea*, with efficacy increasing with concentration and reaching complete inhibition at 10%; because each concentration was analysed in a separate one-way model, this concentration trend is reported descriptively and was not formally tested as a product × concentration interaction. Jerusalem artichoke extract was consistently the most effective agent against *P. lingam* (88–92% inhibition, largely independent of concentration), while *Enterobacter* sp. showed a comparatively narrow spectrum, with no measurable activity against *B. cinerea* and only negligible activity against *S. sclerotiorum* (≤6% inhibition at all tested concentrations). In the greenhouse, all four bioproducts significantly stimulated seedling growth, with fresh shoot weight increases exceeding 100% relative to the untreated control; fresh root weight showed a similar but non-significant trend. Under field conditions, garlic extract and *Paenibacillus* sp. gave the most consistent statistically significant and, in several site-years, fungicide-comparable control of Phoma stem canker (*Phoma lingam*) and Alternaria black spot (*Alternaria* spp.), occasionally matching the fungicide Protikon 250 EC; on the other hand, *Enterobacter* sp. was more variable and Jerusalem artichoke extract intermediate. Seed yield was increased over the untreated control at Rzeszów in both seasons, with the exception of *Enterobacter* sp. in 2023/2024, but not at Winna Góra, and thousand-seed weight was unaffected by treatment.

## 1. Introduction

Winter oilseed rape (*Brassica napus* L.) is one of the most important oilseed crops worldwide, and its production can be markedly reduced by a range of fungal diseases affecting leaves, stems and pods throughout the growing season. Among the most damaging pathogens are *Alternaria alternata* (Fr.) Keissl., 1912 and *Alternaria brassicicola* (Schwein.) Wiltshire, 1947, the causal agents of Alternaria black spot; *Botrytis cinerea* Pers., 1801, the causal agent of grey mould; *Phoma lingam* (Tode) Desm., 1849 (teleomorph *Leptosphaeria maculans* (Desm.) Ces. & De Not., 1863, currently reclassified in the genus *Plenodomus* as *Plenodomus lingam* (Tode) Höhn., 1911; the closely related *P. biglobosus* also occurs on oilseed rape), which causes Phoma stem canker (blackleg) of crucifers; and *Sclerotinia sclerotiorum* (Lib.) de Bary, 1884, the causal agent of sclerotinia stem rot. For consistency with the field assessment methodology applied in this study, the anamorph name *Phoma lingam* is used throughout; subsequent mentions of these species are abbreviated (e.g., *A. alternata*, *A. brassicicola*, *B. cinerea*, *S. sclerotiorum*). The economic stakes are considerable: *Phoma* stem canker alone causes annual losses estimated at over £56 million per season in England and more than AUD 100 million per season in Australia even where fungicides are routinely applied, and is regarded as one of the most damaging diseases of oilseed rape worldwide [1], while sclerotinia stem rot typically reduces the yield of affected crops by 10–20%, with losses of up to 50% reported in heavily infested fields [2].

Chemical control of these pathogens currently relies mainly on synthetic fungicides, in particular triazole compounds such as prothioconazole. Concerns about the environmental persistence of synthetic fungicides, the development of pathogen resistance and non-target effects have intensified interest in biological and plant-derived alternatives that can be integrated into rapeseed protection programmes.

Garlic (*Allium sativum* L.) extracts contain organosulphur compounds, most notably allicin, which display broad-spectrum antimicrobial activity against plant pathogenic fungi, bacteria and oomycetes [3,4]. Garlic extracts and essential oils have repeatedly been shown to inhibit the mycelial growth of *B. cinerea*, *Penicillium expansum* Link, 1809 and *Neofabraea alba* Guthrie, 1959 [5], as well as a wide range of other fungi, including *Fusarium*, *Alternaria* and *Aspergillus* spp. [6,7].

Jerusalem artichoke (*Helianthus tuberosus* L.), a crop already cultivated for tubers and biomass, also accumulates phenolic compounds in its leaves that possess documented antifungal activity [8,9]. Leaf extracts of *H. tuberosus* have been reported to inhibit mycelial growth of several phytopathogenic fungi, including *B. cinerea*, *Rhizoctonia solani* J.G. Kühn, 1858 and *Alternaria solani* Sorauer, 1896 [10,11], while spontaneous fermentation of Jerusalem artichoke juice has been shown to suppress *Fusarium* and *Alternaria* [12].

Plant growth-promoting rhizobacteria (PGPR), in particular species of *Paenibacillus* and *Enterobacter*, have proved to be effective fungistatic agents through multiple mechanisms, including production of antibiotics and hydrolytic enzymes, competition, and induction of systemic resistance [13,14]. *Enterobacter* agglomerans, for example, produces the antibiotic pyrrolnitrin, active against *Alternaria* spp., *B. cinerea* and *R. solani* [15], while *Paenibacillus polymyxa* (Prazmowski) Ash, Priest & Collins, 1994 produces fusaricidin-type lipopeptide antibiotics active against *Leptosphaeria maculans*, the causal agent of blackleg of canola [16].

The aim of this study was to evaluate the effect of two plant extracts, from garlic (*Allium sativum* L., 1753) and Jerusalem artichoke (*Helianthus tuberosus* L., 1753), and two bacterial fermentation supernatants (*Enterobacter* sp. and *Paenibacillus* sp.), in limiting the growth of important oilseed rape pathogens under in vitro conditions, stimulating rapeseed development under greenhouse conditions, and controlling pathogens and affecting yield under field conditions. The chemical profiles of the preparations used here were characterised previously in the context of cereal pathogens [17]; the present work extends their evaluation to winter oilseed rape and, for the first time, to multi-season, two-site field conditions with seed yield and thousand-seed weight endpoints. To our knowledge, this is the first study to evaluate the field efficacy of these two plant extracts and two bacterial supernatants against oilseed rape pathogens across two contrasting locations and two growing seasons, and specifically to examine, through a formal three-way (Treatment × Location × Season) analysis of variance, the extent to which field efficacy is consistent across environments or instead depends on site- and season-specific conditions—an aspect that is frequently asserted but rarely tested statistically in comparable biocontrol studies.

## 2. Results

### 2.1. Chemical Characterisation of the Tested Bioproducts

Chemical profiling of the plant extracts, performed on the same preparation batches used throughout this study, showed a total phenolic content (expressed as mg gallic acid equivalents per 100 g of extract, mg GAE/100 g of extract) of 595.7 ± 15.4 mg GAE/100 g in the Jerusalem artichoke extract versus 507.2 ± 12.4 mg GAE/100 g in the garlic extract; this difference was statistically significant (*p* < 0.05, Tukey’s HSD test, as reported in the companion characterisation study [17]). Qualitative screening additionally detected tannins in the Jerusalem artichoke extract but not in the garlic extract, while both extracts contained saponins and flavonoids. The garlic extract additionally contained allicin at 83.3 ± 0.3 mg/L and betulin, its dominant triterpene, at 275.1 ± 0.03 mg/L (*n* = 2 analytical replicates); its elemental profile showed K, P, S, Ca and Mg as the major macro-elements (Table 1), with Hg, Cr, Ni, Pb and Co below the detection limit for the analytical method used [17]; B-group vitamins and vitamin E isoforms were also detected, each below 30 mg/L.

HPLC analysis of the two fermentation supernatants revealed distinct metabolic fingerprints: the *Enterobacter* sp. broth was dominated by glycerol, 2,3-butanediol and acetic acid, whereas the *Paenibacillus* sp. broth contained these same compounds plus lactic acid, ethanol and succinic acid, indicating a more diverse metabolite profile (the relationship between this broader profile and antifungal activity is examined further in Section 3). Full quantitative data, including the complete elemental, vitamin, amino acid and organic acid profiles, are reported in the companion characterisation study [17], which used the same product batches to evaluate activity against cereal pathogens. The corresponding summary values are compiled in Table 1. The pH of the two supernatants was not determined in the original characterisation study; given the substantial organic acid content of both broths (up to ca. 7–8 g/L glycerol and 1–3 g/L organic acids, [17]), a contribution of medium acidification to the in vitro antifungal activity reported below cannot be excluded, and pH measurement of the supernatants together with a pH-matched or neutralised-supernatant control are recommended in future work to disentangle acidification from specific antimicrobial metabolite effects.

Values are means ± SD as reported in [17]. Complete compositional data—including the full elemental profile, individually quantified amino acids, triterpenes, polyphenols and vitamins, and the HPLC chromatograms and calibration parameters of the fermentation supernatants—are available therein. Data summarised from [17]. GAE—gallic acid equivalents.

### 2.2. In Vitro Results

The tested bioproducts showed differentiated activity in limiting mycelial growth of the tested pathogens, depending on the product, its concentration and the pathogen species (Table 2). The comparator product, containing the active substance prothioconazole, inhibited mycelial growth of all tested pathogens by 100%. The *Paenibacillus* sp. supernatant showed, on average, the highest activity with the broadest spectrum, reaching inhibition levels comparable to prothioconazole against most pathogens, in particular *A. brassicicola* and *B. cinerea*. Jerusalem artichoke extract was the most effective agent against *P. lingam*, while garlic extract stood out for its inhibition of *S. sclerotiorum* and *B. cinerea* mycelial growth.

At 1% concentration, the highest efficacy was recorded for *Paenibacillus* sp. against *A. brassicicola* (87.6%) and *B. cinerea* (89.1%). Jerusalem artichoke was most effective against *P. lingam* (88.3%). At 5%, garlic extract achieved its highest efficacy against *S. sclerotiorum* (96.9%), and *Paenibacillus* sp. reached 97.6% inhibition of *B. cinerea*. At 10%, both garlic extract and *Paenibacillus* sp. reached 100% inhibition of *B. cinerea*; against *S. sclerotiorum* at 10%, garlic extract also reached 100% inhibition, while *Paenibacillus* sp. reached a somewhat lower, though still high, 82.8% inhibition. Jerusalem artichoke retained a high efficacy against *P. lingam* at 10% (88.1%).

Considering the overall performance of the individual bioproducts, *Paenibacillus* sp. showed the broadest spectrum of activity and high efficacy already at the lowest concentration tested (1%), being particularly effective against *B. cinerea* (89–100%) and *A. brassicicola* (82–88%). Garlic extract showed a strong concentration dependence: at 10% it reached 100% inhibition of *B. cinerea* and *S. sclerotiorum*, while at 1% its efficacy was considerably lower. Jerusalem artichoke was characterised by stable, high efficacy against *P. lingam* (88–92%) largely independent of concentration, while it performed poorly against *S. sclerotiorum*. *Enterobacter* sp. had a comparatively narrow spectrum of activity, showing no inhibitory activity against *B. cinerea* at any tested concentration and only negligible activity against *S. sclerotiorum* (0% at 1% and 5%, rising to 6.3% at 10%), together with medium inhibitory activity against *P. lingam* (56–73%).

### 2.3. Greenhouse Results

**Figure 1:**
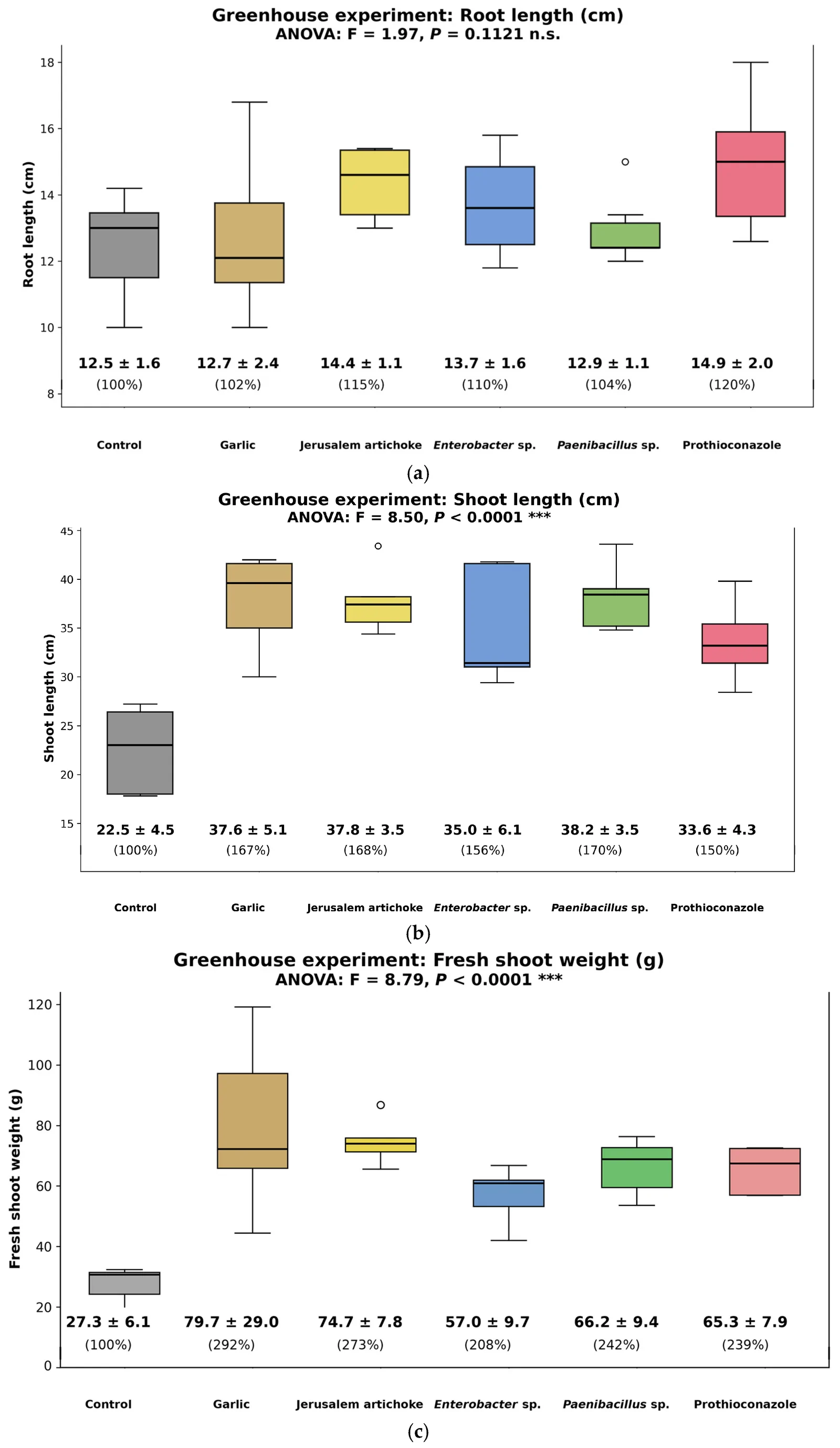

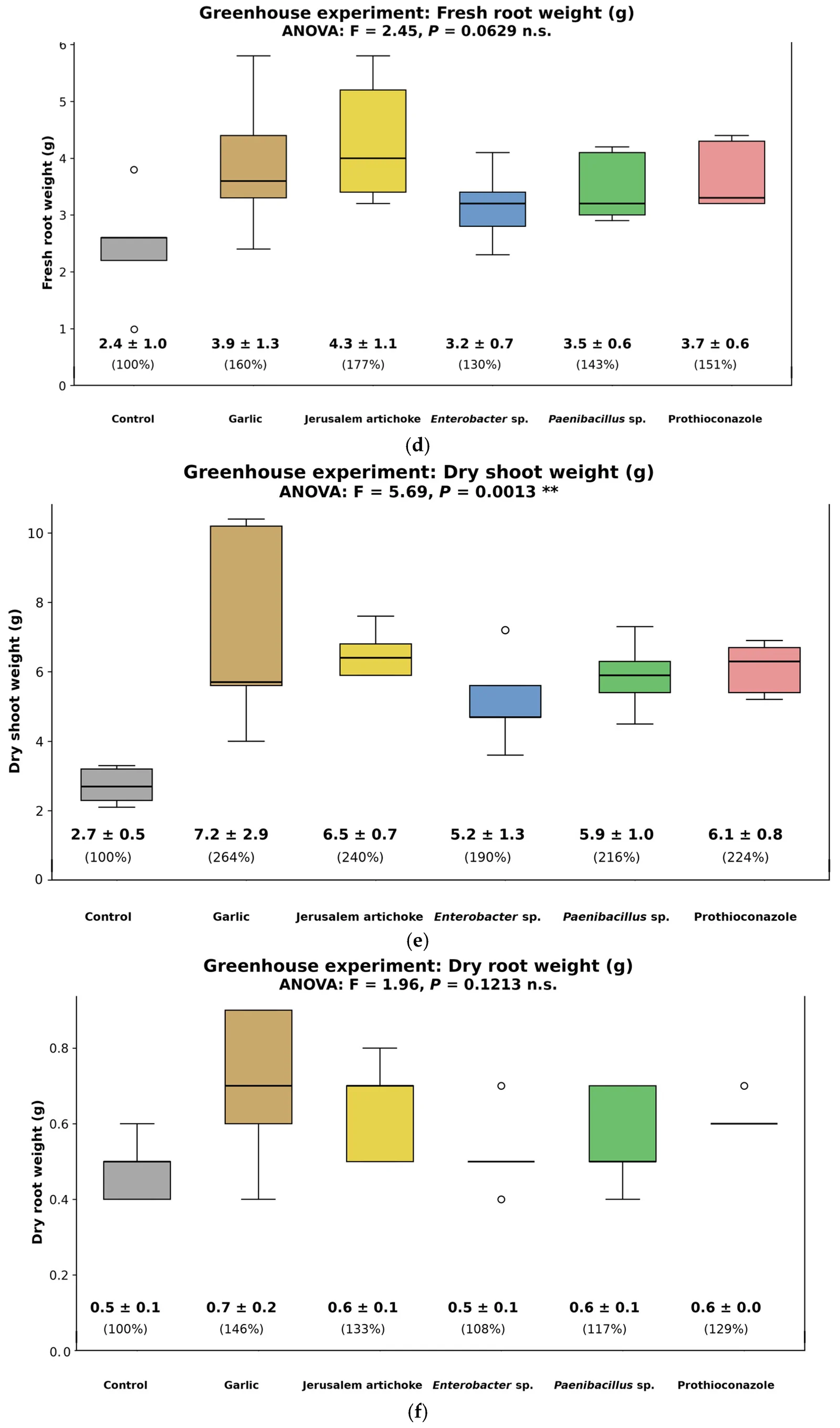
Effect of the biological preparations on growth parameters of winter oilseed rape seedlings in the greenhouse experiment: (**a**) root length (cm); (**b**) shoot length (cm); (**c**) fresh shoot weight (g); (**d**) fresh root weight (g); (**e**) dry shoot weight (g); (**f**) dry root weight (g). Values below the boxes are means ± SD; values in parentheses are expressed as a percentage of the untreated control. ANOVA significance: *** *p* < 0.001, ** *p* < 0.01, n.s.—not significant. Circles indicate outliers.

In the greenhouse experiments, all tested products stimulated plant growth. A significant increase was observed for shoot length, fresh shoot weight and dry shoot weight relative to the untreated control; fresh root weight showed a positive but non-significant trend (*p* = 0.0629; Figure 1d). All biological preparations induced marked stimulation of plant growth, with shoot biomass increases exceeding 100% compared with the untreated control (Table 3, Figure 1).

### 2.4. Efficacy Profiles

**Figure 2:**
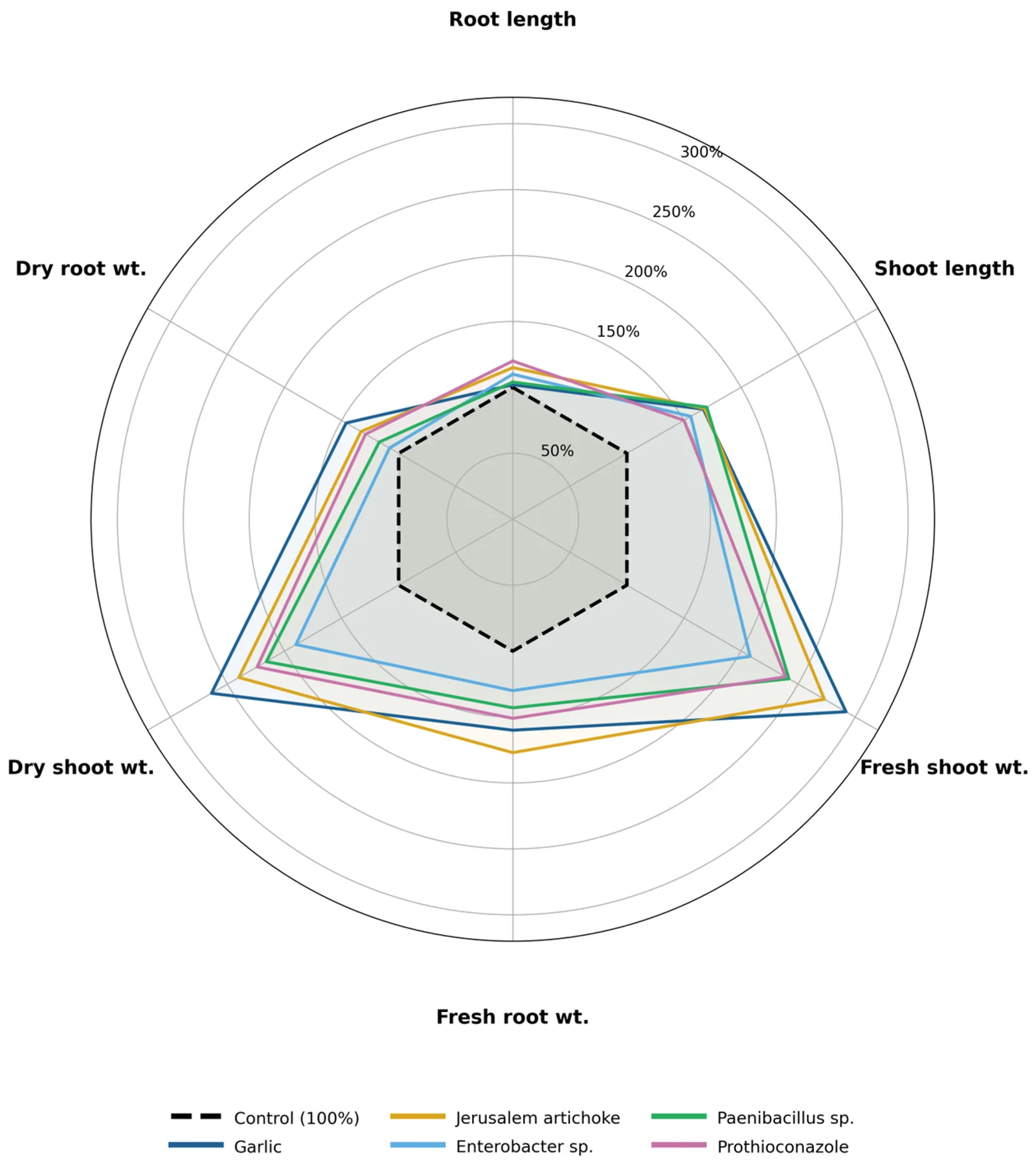
Efficacy profile of the tested combinations in the greenhouse experiment.

Radar plot analysis revealed distinct activity profiles for each biocontrol/biostimulation agent (Figure 2). *Paenibacillus* sp. showed a broad biostimulant response across all measured growth parameters, whereas the plant extracts were characterised by pathogen- and parameter-specific patterns of efficacy.

### 2.5. Field Trial Results

Weather conditions differed markedly between the two locations and the two growing seasons during the periods relevant to disease development and to the two spray applications (autumn, BBCH 14–16, and spring regrowth, BBCH 39) (Table 4). In the 2022/2023 season, the autumn period (August–November) was distinctly drier at Rzeszów (84.2 mm total precipitation) than at Winna Góra (139.0 mm). In the 2023/2024 season, autumn precipitation at Rzeszów (data available for September–November only) exceeded that at Winna Góra.

#### 2.5.1. Disease Control

Disease efficacy and yield data for the field trials were subjected to ANOVA followed by Tukey’s HSD post hoc test (see Section 4.5.); different letters within a column in Table 5, Table 6 and Table 7 indicate statistically significant differences between treatments (*p* < 0.05). For the disease efficacy tables, the untreated control column reports mean disease severity (% leaf area affected), while all remaining values are Abbott efficacy (%) relative to that control.

#### 2.5.2. Disease Control at Winna Góra

**Figure 3:**
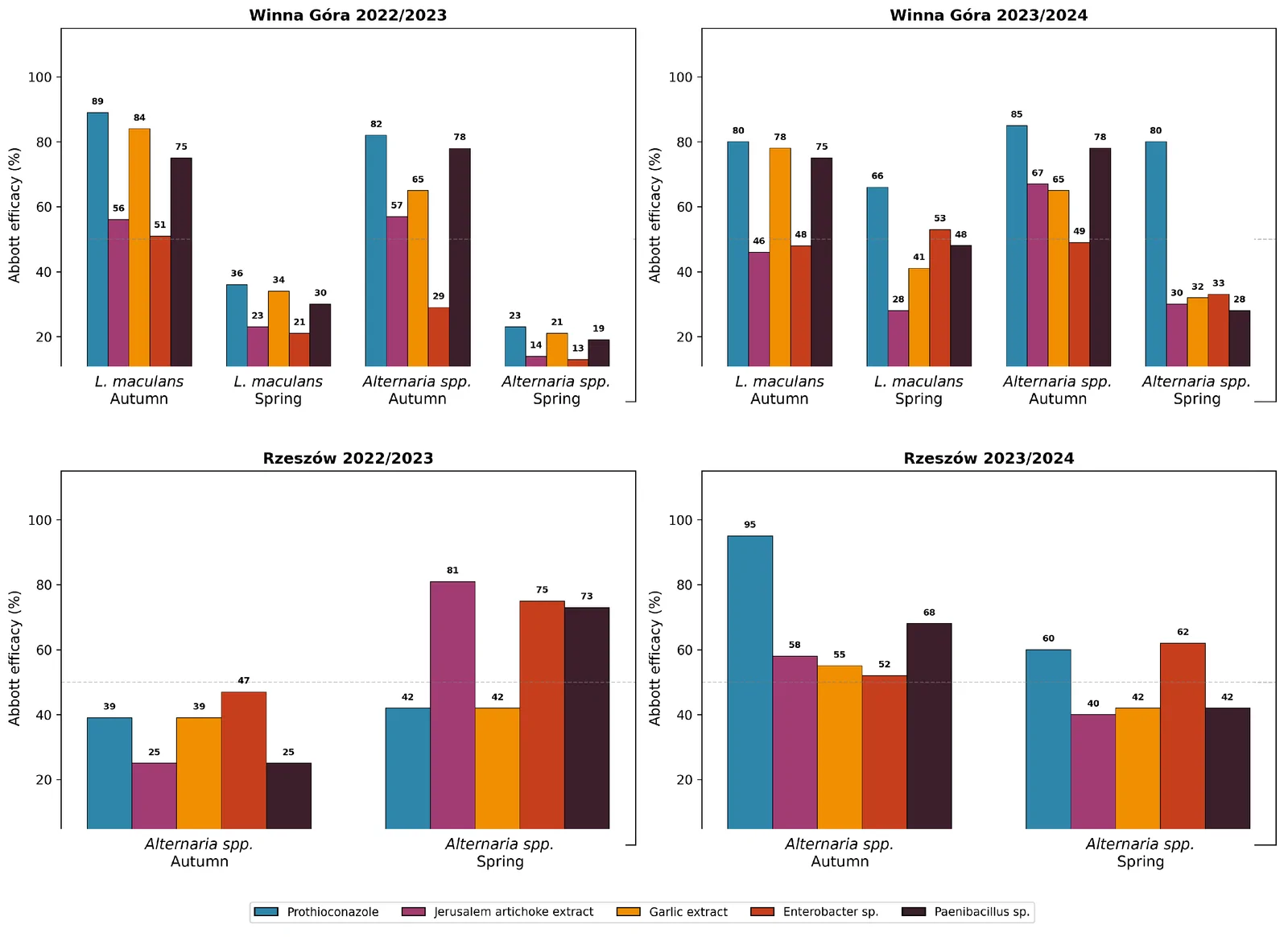
Efficacy of the tested biological preparations and the synthetic fungicide standard (Prothioconazole) against *Phoma* stem canker and *Alternaria* black spot at Winna Góra and Rzeszów, autumn and spring assessments, 2022/2023 and 2023/2024 (Abbott efficacy, %).

At Winna Góra, both Phoma stem canker (*Phoma lingam*) and Alternaria black spot (*Alternaria* spp.) were assessed in both seasons, at both the autumn and spring dates (Table 5, Figure 3). In 2022/2023, autumn Phoma stem canker severity in the untreated control was 15.0%. Prothioconazole and garlic extract gave the highest efficacy, respectively, 89% and 84%, followed by *Paenibacillus* sp. (75%), while Jerusalem artichoke extract and *Enterobacter* sp. were significantly less effective (56% and 51%, respectively). For autumn Alternaria black spot control, severity was measured at 12.0%. Prothioconazole and *Paenibacillus* sp. were statistically equivalent and most effective (82% and 78% efficacy), garlic extract was intermediate (65%). Jerusalem artichoke extract was significantly weaker (57%). *Enterobacter* sp. (29%) did not differ significantly from the untreated control. Efficacy against both diseases declined substantially by the spring assessment for all treatments: only prothioconazole and garlic extract differed significantly from the control for Phoma stem canker, and the same two treatments (prothioconazole, 23%, and garlic extract, 21%) were also the only ones significantly better than the control for Alternaria black spot, with Jerusalem artichoke extract, *Enterobacter* sp. and *Paenibacillus* sp. forming an intermediate, non-significant group relative to the control for that disease.

In 2023/2024, autumn Phoma stem canker severity in the control was 18.0% of infected leaf area. Prothioconazole, garlic extract and *Paenibacillus* sp. formed a statistically equivalent top-performing group (80%, 78% and 75%), while Jerusalem artichoke extract and *Enterobacter* sp. (46% and 48%) were significantly less effective, though still significantly better than the control. At the spring assessment, prothioconazole remained most effective (66%); *Enterobacter* sp. (53%) overlapped with both the top and intermediate groups; garlic extract and *Paenibacillus* sp. (41% and 48%) formed an intermediate group; and Jerusalem artichoke extract (28%) was weakest, not clearly different from the control. For autumn Alternaria black spot (control 14.0% of infected leaf area), prothioconazole and *Paenibacillus* sp. again formed the top group (85% and 78%), garlic extract and Jerusalem artichoke extract were intermediate (65% and 67%), and *Enterobacter* sp. (49%). At the spring assessment, prothioconazole (80%) far exceeded all bioproducts, which formed a single intermediate group (28–33%).

#### 2.5.3. Disease Control at Rzeszów

At Rzeszów, Phoma stem canker (*Phoma lingam*) was not recorded in either season, and only Alternaria black spot (*Alternaria* spp.) efficacy is reported (Table 6, Figure 3). In 2022/2023, autumn severity in the control was 20.0% of infected leaf area. Prothioconazole, garlic extract and *Enterobacter* sp. formed a statistically equivalent group (39%, 39% and 47%), while Jerusalem artichoke extract and *Paenibacillus* sp. (25% each) did not differ significantly from the control. Notably, the ranking reversed at the spring assessment (control severity: 15.0% of infected leaf area). Jerusalem artichoke extract and *Enterobacter* sp. formed the clearest best-performing group (81% and 75%), significantly exceeding prothioconazole and garlic extract (42% each). The only instance in this study where bioproducts significantly outperformed the synthetic fungicide standard. *Paenibacillus* sp. (73%) was numerically similar to this top group, but its overlapping “cd” Tukey grouping with prothioconazole and garlic extract (“bc”, sharing the letter “c”) means it was not statistically distinguishable from either the top group or the fungicide standard (Table 6).

In 2023/2024, autumn severity in the control was 22.0% of infected leaf area. Prothioconazole was by far the most effective treatment (95%) followed by *Paenibacillus* sp. (68%); Jerusalem artichoke extract (58%) overlapped with both the *Paenibacillus* and lowest-performing groups, while garlic extract and *Enterobacter* sp. (55% and 52%) formed the weakest active group. All combinations were significantly effective in comparison to the control object.

At the spring assessment (control severity 12.0% of infected leaf area), prothioconazole and *Enterobacter* sp. formed the top group (60% and 62%) and were significantly better than the control; Jerusalem artichoke extract, garlic extract and *Paenibacillus* sp. (40%, 42% and 42%) were numerically lower and, based on their overlapping “ab” Tukey grouping with the untreated control (“a”, Table 6), were not statistically distinguishable from it.

#### 2.5.4. Yield and Thousand-Seed Weight

Seed yield (t/ha) and thousand-seed weight (TSW, g) of winter oilseed rape cv. Artemis were recorded at both locations in both seasons (Table 7).

Seed yield of winter oilseed rape cv. Artemis depended clearly on the site and season, and to a lesser extent on the treatment applied (Table 7). The most productive conditions occurred at Rzeszów in 2022/2023, where yields reached up to 5.07 t/ha, while the poorest were recorded at Winna Góra in 2023/2024 (2.55–2.80 t/ha), reflecting a difficult season at that location.

The response to treatment was most evident at Rzeszów. In both seasons, most treatments improved yield relative to the untreated control, which remained the lowest or among the lowest-yielding in each case; the exception was *Enterobacter* sp. in 2023/2024, whose yield (3.54 t/ha) was not statistically distinguishable from the control (3.32 t/ha), based on overlapping Tukey groupings (Table 7). Prothioconazole performed best, raising yield from 4.36 to 5.07 t/ha in 2022/2023 and from 3.32 to 4.29 t/ha in 2023/2024—a gain of roughly one tonne per hectare over the control. The plant extracts and bacterial treatments gave intermediate yields, generally exceeding the control but not reaching the level of prothioconazole.

At Winna Góra the picture was different. In 2022/2023, the treatments did not improve on the untreated control (4.16 t/ha); here prothioconazole again gave the highest yield (4.27 t/ha), but the garlic extract and *Enterobacter* sp. (3.94 and 3.93 t/ha) were the lowest and differed significantly only from prothioconazole. In the following, low-yielding season, the treatments produced comparable yields, with no clear differences between them.

Thousand-seed weight proved far more stable. It varied only within a narrow range (4.22–5.19 g) and was not affected by any treatment at either site or in either season. The yield gains observed at Rzeszów therefore did not result from heavier seeds; a possible, though unmeasured, explanation is that they instead reflect other yield components such as the number of seeds set per plant, which was not recorded in this study.

To formally test whether treatment efficacy and yield response were consistent across locations and seasons, rather than remaining a purely descriptive observation, the field disease severity, seed yield and thousand-seed-weight data were additionally analysed using combined three-way factorial ANOVA models (Type II sums of squares; Table 8, Table 9 and Table 10). For seed yield, Treatment was significant (*p* = 0.001) but explained only 3.3% of the total variance, whereas Location (η^2^ = 25.1%) and Season (η^2^ = 41.9%) dominated, with a significant Location × Season interaction; none of the interactions involving Treatment (Treatment × Location, *p* = 0.083; Treatment × Season; Treatment × Location × Season) was significant, and no term involving Treatment was significant for thousand-seed weight (Table 8). For *Alternaria* black spot severity, Treatment was the dominant source of variation (η^2^ = 56.6%), and the Treatment × Location, Treatment × Season and Treatment × Location × Season interactions were all significant (*p* < 0.001), formally confirming that the relative efficacy of the treatments differed between sites and seasons (Table 9). For *Phoma* stem canker at Winna Góra, Treatment again dominated (η^2^ = 65.4%), with significant Treatment × Season (*p* = 0.020) and Treatment × Observation time (*p* < 0.001) interactions—the latter reflecting the general decline in efficacy between the autumn and spring assessments—while the three-way interaction was not significant (Table 10). This combined analysis is presented in addition to, not instead of, the within-location–season one-way ANOVAs reported in Table 5, Table 6 and Table 7.

## 3. Discussion

The broader metabolic profile of the *Paenibacillus* sp. supernatant relative to *Enterobacter* sp.—notably the additional presence of lactic acid, ethanol and succinic acid alongside glycerol and 2,3-butanediol—occurs alongside the broader-spectrum antifungal activity of the *Paenibacillus* sp. supernatant observed against the rapeseed pathogens tested here. We note this association is correlative: the metabolites quantified by HPLC were not individually tested for antifungal activity, and no causal relationship between the broader metabolite profile and the wider biocontrol spectrum was demonstrated in this study. Likewise, the comparatively high phenolic content and the additional presence of tannins in the Jerusalem artichoke extract, relative to the allicin- and betulin-dominated profile of the garlic extract, are compatible with, but do not by themselves establish, the largely non-overlapping pathogen spectra of the two plant extracts.

The pronounced, broad-spectrum antifungal activity of the *Paenibacillus* sp. supernatant is consistent with earlier reports documenting the production of numerous antimicrobial compounds by this genus. *Paenibacillus* spp. are known to produce lipopeptide antibiotics, including fusaricidins and polymyxins, which disrupt fungal membrane integrity [13,16]; the effectiveness of fusaricidin-type antibiotics of *P. polymyxa* against *Leptosphaeria maculans* (the teleomorph of *Phoma lingam*) has been demonstrated directly in other studies [16,18,19,20]. We emphasise, however, that fusaricidins, polymyxins and the other metabolites discussed below were not analytically confirmed in the specific *Paenibacillus* sp. supernatant tested here (only glycerol, 2,3-butanediol, acetic, lactic and succinic acid and ethanol were quantified by HPLC, [17]); the following comparisons with the literature are therefore offered as plausible mechanistic context rather than as evidence that these particular compounds caused the antifungal activity observed in the present study. In addition, hydrolytic enzymes such as chitinases and β-1,3-glucanases contribute to fungal cell wall degradation in other *Paenibacillus* strains [13,21], and expression of a *P. polymyxa* β-1,3-1,4-glucanase in *Streptomyces lydicus* has been shown to improve biocontrol of *B. cinerea* [22]. Small antifungal metabolites such as methyl 2,3-dihydroxybenzoate and protocatechuic acid, isolated from *Paenibacillus elgii* HOA73, have likewise shown strong activity against *B. cinerea* and *R. solani* [23,24], and field scale control of grey mould of tomato by *P. elgii* HOA73 has been reported [25], together with control of tomato grey mould by *P. peoriae* BC-39 [26]. Similarly broad antagonism, including strong activity against *A. alternata*, *B. cinerea* and *Fusarium oxysporum*, has been described for *P. alvei* DZ-3 [27], directly agreeing with the breadth of activity we observed for our *Paenibacillus* sp. supernatant against *A. brassicicola* and *B. cinerea*, although DZ-3 was additionally active against *F. oxysporum*, a pathogen not included in our panel, so the two studies are complementary rather than directly overlapping in pathogen scope. Two further comparisons illustrate an important divergence rather than a straightforward agreement: *P. alvei* K165 reduced *S. sclerotiorum*, *R. solani* and *Pythium ultimum* on lettuce specifically through induction of systemic resistance in the host plant [28], and *P. ehimensis* showed strong chitinase/glucanase-dependent activity against *A. alternata* that required continued enzyme production by living cells [21]. Both mechanisms—induced systemic resistance and de novo enzyme production—depend on a metabolically active, plant- or medium-associated bacterial population, which is not present in the cell-free fermentation supernatant tested in the present study (Section 4.1); any contribution of comparable enzymes to our results could only occur through activity already present in the harvested broth at the time of application, a more limited pathway than the continuous production described in [21,28]. This distinction may help explain why our *Paenibacillus* sp. supernatant, despite its broad in vitro spectrum, showed a comparatively modest, though still measurable, growth-promotion and disease-control profile in the greenhouse and field relative to what live-cell inoculation studies such as [21,28] report, and it is a direct illustration of the general caveat, raised throughout this discussion, that supernatant-only preparations cannot be assumed to reproduce every mechanism documented for the corresponding live bacterium. A recent review supports the broader relevance of this comparison, confirming that, beyond *P. polymyxa*, multiple *Paenibacillus* species—consistent with the present, taxonomically unresolved *Paenibacillus* sp. Isolate—merit attention as biocontrol agents against fungal phytopathogens [14].

The pathogen-specific activity patterns observed for the plant extracts reflect differences in their bioactive compound profiles. In the present study, garlic extract showed outstanding activity against *S. sclerotiorum* and *B. cinerea*, consistent with earlier reports on the activity of allicin and garlic extracts/essential oils against these pathogens [3,5,29,30,31]. This agreement extends to other crop systems: garlic extract has similarly reduced *Fusarium* and *S. sclerotiorum* severity on potato [32] and inhibited *F. oxysporum* f. sp. *ciceri* and *S. sclerotiorum* [33], and has shown measurable, though comparatively modest, activity against *S. sclerotiorum* among other pepper pathogens [34]; the magnitude of inhibition we observed for *S. sclerotiorum* (up to 100% at 10%) was numerically higher than reported in [34], which may reflect differences in extract concentration, preparation method (aqueous versus other solvent systems) or pathogen isolate, but the overall pattern—garlic extract as a comparatively strong option specifically against *Sclerotinia*/*Botrytis*-type pathogens across several unrelated crops (potato [32], chickpea [33], pepper [34], and rapeseed here)—is directly complementary across host systems rather than being contradicted by any of these three studies. By contrast, the garlic extract used here was markedly less effective against *Alternaria alternata* and, at the lowest concentration, *A. brassicicola*, than several previous studies would suggest, where garlic extracts have reduced *Alternaria* leaf spot severity on tomato, coriander, mung bean, faba bean and Roselle, and inhibited *Alternaria* and *Fusarium* spp. in vitro [7,35,36,37,38,39,40,41,42]; this discrepancy, in contrast to the good agreement seen for *S. sclerotiorum*/*B. cinerea*, may reflect differences in extraction method, garlic cultivar and allicin content, and in pathogen isolate sensitivity, and points to a need for further optimisation of garlic-based products specifically against *Alternaria* spp. of rapeseed.

Jerusalem artichoke extract showed outstanding, and largely novel, efficacy against *P. lingam*, with potential applications in the control of the causal agent of Phoma stem canker of crucifers. This finding is consistent with the broad antifungal activity reported for *H. tuberosus* leaf extracts, which have inhibited nine phytopathogenic fungi by 16.9–98.2%, with particularly strong effects against *B. cinerea*, *Colletotrichum gloeosporioides* (Penz.) Penz. & Sacc., 1884, *Phytophthora capsici* Leonian, 1922 and *Rhizoctonia cerealis* E.P. Hoeven, 1977 [8], and with reports of good efficacy against *Rhizoctonia solani* J.G. Kühn, 1858, *Alternaria solani* Sorauer, 1896 and *B. cinerea* [10], strong inhibition of grey mould of pepper by acetone leaf extracts [11], and inhibition of *S. sclerotiorum* and *P. capsici* by phenolic acids isolated from *H. tuberosus* leaves [43]. The antifungal activity of *H. tuberosus* extracts has been attributed to their phenolic content [9], and fungicidal/bactericidal activity has also been reported for supercritical extracts of the aerial parts of *H. tuberosus* [44], while fermentation of Jerusalem artichoke juice has been shown to suppress *Fusarium* and *Alternaria* [12,17].

The complete absence of activity of the *Enterobacter* sp. supernatant against *B. cinerea* and *S. sclerotiorum* at all tested concentrations is notable, given the numerous reports of *Enterobacter* spp. acting as effective antagonists of these two pathogens: *E. agglomerans* (via pyrrolnitrin), *E. aerogenes*, *E. cloacae*, *E. cowanii*, *E. roggenkampii* and *E. intermedius* have all been reported to antagonise *B. cinerea* [15,45,46,47,48,49,50,51], and *E. aerogenes*, *E. cloacae* and *Enterobacter* sp. BA48R have reduced *S. sclerotiorum* growth or symptoms [45,52,53], while bacterial endophytes including *Enterobacter* have reduced *Sclerotinia* disease specifically on oilseed rape [54]. The lack of inhibition observed here for the *Enterobacter* sp. fermentation supernatant, as opposed to live-cell antagonism used in most of the cited studies, suggests that the active antifungal metabolites relevant to these two pathogens may not accumulate, or may be inactivated, under the fermentation conditions used to prepare the tested product, or that the strain used lacks the corresponding biosynthetic pathways. By contrast, *Enterobacter* sp. did show clear activity against *P. lingam* and moderate activity against *Alternaria* spp., in line with reports of *E. roggenkampii*, *E. cloacae*, *E. asburiae*, *E. chengduensis* and *Enterobacter* sp. inhibiting *Alternaria alternata* [55,56,57,58,59].

The consistent, strong growth stimulation observed across all four bioproducts (Section 2.3) is compatible with the plant growth-promoting properties reported for the genera tested. *Enterobacter roggenkampii* Sutton has been characterised as a nitrogen-fixing, plant growth-promoting endophytic bacterium combining biocontrol and stress-tolerance properties [48]; however, that characterisation refers to viable, root-colonising cells, whereas the product tested here was a cell-free fermentation supernatant. The pronounced stimulation of shoot and root biomass observed for the *Enterobacter* sp. supernatant despite its narrow antifungal spectrum in vitro is therefore more plausibly attributed to extracellular metabolites carried over in the supernatant (e.g., glycerol, 2,3-butanediol, organic acids, and possibly diffusible phytohormones or siderophores [17]), than to *in planta* nitrogen fixation or other activities that require living bacterial cells; biological nitrogen fixation as a mechanism is not directly applicable to a sterile, cell-free preparation and is not proposed as an explanation for the growth response observed here.

Across both locations and seasons, garlic extract and *Paenibacillus* sp. were consistently among the best-performing bioproducts against Phoma stem canker at Winna Góra, mirroring the strong in vitro activity of *Paenibacillus* sp. supernatant against *P. lingam* and the documented activity of *Paenibacillus* polymyxa fusaricidins against Leptosphaeria maculans [16,18,19]. Enterobacter sp. showed the most variable performance of the four bioproducts, ranging from not significantly different from the untreated control (Winna Góra, autumn Alternaria 2022/2023; Rzeszów, autumn 2022/2023) to among the most effective treatments at other assessment dates, particularly the spring assessment at Rzeszów, consistent with the comparatively narrow but pathogen-dependent spectrum already noted in vitro. Jerusalem artichoke extract, by contrast, was among the weakest bioproducts against Phoma stem canker at Winna Góra in both seasons (46–56% efficacy, generally the lowest or second-lowest of the four bioproducts), despite being the standout performer against *P. lingam* in vitro (88–92% inhibition, largely independent of concentration). This in vitro-to-field discrepancy contrasts with the otherwise good agreement seen for garlic extract and *Paenibacillus* sp. We had previously attributed this discrepancy to lower field persistence of the phenolic compounds of *H. tuberosus* extract relative to the sulphur compounds of garlic or the lipopeptide antibiotics of *Paenibacillus* sp.; however, this explanation should be treated with caution, since direct photostability data specifically indicate that chlorogenic acid—a major phenolic constituent of *Helianthus tuberosus* leaves—is not degraded by UVA or UVB irradiation in solution [60], which argues against simple UV photodegradation as the mechanism and against a straightforward comparison with the chemical instability of allicin, which is well documented and driven principally by enzymatic and thermal, rather than photochemical, factors. Reduced field efficacy of the Jerusalem artichoke extract may instead reflect other routes of loss under field conditions not tested here, such as rain wash-off, microbial or enzymatic degradation on the leaf surface, oxidation, or reduced bioavailability/uptake relative to the in vitro assay, and we present this as a plausible but unconfirmed explanation requiring dedicated field persistence studies. Jerusalem artichoke extract performed more competitively against Alternaria black spot, and was, jointly with *Enterobacter* sp. and *Paenibacillus* sp., among the treatments that numerically exceeded the fungicide standard at Rzeszów in spring 2022/2023 (Table 6)—the only instance in this study where several bioproducts outperformed Protikon 250 EC on a numerical basis, although it should be noted that not all of these pairwise differences were statistically distinguishable from the fungicide standard (overlapping statistical groupings, Table 6).

Taken together, the field data indicate that the tested bioproducts, and particularly garlic extract and *Paenibacillus* sp., produced statistically significant and, in several site-years, fungicide-comparable reductions in Phoma stem canker and Alternaria black spot severity. At Rzeszów, these effects were accompanied by significant increases in seed yield over the untreated control in both seasons, where all or most bioproducts out-yielded the control, whereas at Winna Góra the same treatments did not raise yield above the control and, in 2022/2023, ranked among the lowest-yielding. Thousand-seed weight remained unaffected. The yield benefit was thus site-dependent—expressed under the more favourable conditions at Rzeszów but absent at Winna Góra, where the difficult 2023/2024 season in particular masked any treatment effect. This site-specific translation of disease control into yield is consistent with the known capacity of winter oilseed rape to compensate for moderate foliar disease under favourable conditions, and underscores that yield-based conclusions should be drawn cautiously pending further multi-year confirmation.

## 4. Materials and Methods

### 4.1. Chemical Characterisation of Plant Extracts and Bacterial Supernatants

The garlic and Jerusalem artichoke extracts and the *Enterobacter* sp. and *Paenibacillus* sp. fermentation supernatants evaluated in this study were produced from the same preparation batches characterised, in parallel, within the wider FertiUp project for their activity against cereal pathogens [17]; the complete analytical protocols are described there. Briefly, total phenolic content of the plant extracts was determined spectrophotometrically with the Folin–Ciocalteu reagent (Sigma-Aldrich, Merck KGaA, Darmstadt, Germany), using triplicate analyses (*n* = 3) referenced against a gallic acid calibration curve, and their qualitative phytochemical profile (saponins, tannins, flavonoids, terpenoids, alkaloids, coumarins, cardiac glycosides, anthraquinones, phlobatannins and carbohydrates) was screened using standard colorimetric and precipitation assays. The garlic extract was additionally profiled by UHPLC-DAD/FLD (C18 columns; DAD detection at 205–254 nm, FLD at excitation/emission 295–329/330–472 nm; column temperature 20–50 °C; flow rates 0.15–0.64 mL/min, isocratic or gradient elution with water/acid, methanol or acetonitrile mobile phases depending on analyte) and by LC-ESI-MS/MS (MRM mode) for allicin, free amino acids, triterpenes, polyphenols and B-group and lipophilic vitamins (*n* = 2 analytical replicates per analyte, external calibration, solvent blanks included), and by ICP-OES for its elemental composition. The two bacterial fermentation supernatants were analysed by HPLC with refractive index detection (anion exchange column; mobile phase 0.03 M sulphuric acid, 50 °C) to quantify their organic acid and polyol content, using external calibration curves (*R*^2^ = 0.999 for all analytes; triplicate calibration points). Both plant extracts were prepared by ultrasound-assisted extraction (garlic: water extract of the bulb, drug extract ratio 1:2.5; Jerusalem artichoke: water extract of the leaves, drug extract ratio 1:9; both extracted for 1 h at 60 °C) [17]. The two bacterial preparations tested throughout this study (in vitro, greenhouse and field) were cell-free fermentation broths (supernatants) obtained by centrifugation (8000× *g*; 20 min; 4 °C) of the *Enterobacter* sp. and *Paenibacillus* sp. bioreactor cultures followed by filtration using 0.22 µm cellulose filters; they did not contain viable bacterial cells at the point of application (which was confirmed using agar plate culture method on BHI medium), and biological effects reported below should accordingly be attributed to soluble metabolites carried over in the supernatant rather than to living bacterial inoculation. The *Enterobacter* sp. and *Paenibacillus* sp. strains used to generate the supernatants were identified to genus level using matrix-assisted laser desorption/ionisation–time-of-flight–mass spectrometry (MALDI-TOF MS) (Axima Confidence-Shimadzu Corporation, Kyoto, Japan) according to methodology described in [61] and spectrometer manufacturer’s recommendations. The identification of unknown bacteria was performed by comparing protein mass spectral patterns with the Shimadzu Axima Confidence plus SARAMIS microorganism database. Only the summary results relevant to interpreting the present rapeseed bioassays are reported here (Section 2.1); the full quantitative dataset, including complete elemental, vitamin, amino acid and organic acid profiles, is presented in reference [17].

### 4.2. In Vitro Experiments

The mycelial growth of selected oilseed rape pathogens was evaluated in vitro following treatment with the tested products. Test material comprised extracts from two plant species (garlic—*Allium sativum*; Jerusalem artichoke—*Helianthus tuberosus*), two bacterial fermentation supernatants (*Enterobacter* sp. and *Paenibacillus* sp.), one synthetic fungicide active substance (prothioconazole, 250 g/L), and isolates of five pathogenic fungi obtained from rapeseed plant material (Table 11 and Table 12). The most pathogenic available isolates were selected for the study. Plant fragments showing disease symptoms were, after preliminary macroscopic identification, placed on PDA medium. Fungal colonies growing out of the plant material were sub-cultured and further incubated. Fungi were identified based on colony and conidial morphology, using available mycological keys.

Plant and bacterial products were tested at three concentrations (1, 5 and 10% *v*/*v* of the preparation), while the synthetic fungicide was tested at the field application rate. Because the tested plant extracts and bacterial supernatants are complex, multi-component preparations rather than single defined active substances, the 1, 5 and 10% concentrations refer to the proportion (*v*/*v*) of each preparation in the medium and not to an equivalent concentration of a specific active compound; comparisons between preparations at the same nominal % should accordingly be interpreted as comparisons between formulations rather than between equimolar or equivalent active ingredient doses, given their differing drug extract ratios and compositions (Table 12). The tested agents were added to sterile potato dextrose agar (PDA) medium cooled to 45 °C, in amounts giving the target concentration of the preparation. The medium product mixture was poured into Petri dishes. The experiment was set up in five replicates. The control consisted of plain PDA medium without any additive. A 5 mm mycelial plug of each fungal culture was placed in the centre of the solidified medium. Plates were incubated at 20 °C under controlled conditions in a Binder chamber (Sanyo Electric Japan—Incubator MIR-254, PHC Europe BV, Etten-Leur, The Netherlands). Incubation conditions were 20 °C for 14 h during the day and 14 °C for 10 h at night, at 50% humidity. Colony diameter in each combination was measured once the mycelium of the control had overgrown the medium surface. Mean mycelial growth (mm) was calculated, and the percentage inhibition of mycelial growth was calculated using the formula Ow = [(K − F)/K] × 100, where K is control growth and F is growth in the treated combination. All experiments were performed in two independent series, and the series-level mean was used as the experimental unit; the series term was not included as an explicit factor in the ANOVA model. Percentage inhibition of mycelial growth was subjected to one-way analysis of variance (ANOVA) with treatment as the sole factor, run separately for each pathogen and each concentration; where the ANOVA was significant, means were separated using Tukey’s honestly significant difference (HSD) test at *p* < 0.05.

### 4.3. Greenhouse Experiments

The effect of the plant extracts and bacterial supernatants on the development of oilseed rape cv. Artemis was assessed under controlled greenhouse conditions. Rapeseed seeds (10 per pot) were sown into 20 cm diameter pots filled with Kronen sowing substrate (pH 6.0–6.5). The experiment was conducted in four replicate pots per treatment over two series, and the pot (not the individual plant) was treated as the experimental unit for statistical analysis; the five plants sampled per pot (see Section 4.3 below) were averaged to a single pot-level value prior to ANOVA rather than being treated as independent replicates. Two weeks after emergence, the tested plant extracts (10%) and bacterial supernatants (1%) were applied as a foliar spray (Table 12). Each series also included an untreated control. The dose of the reference fungicide was calculated per hectare (per 200 L of water).

The experiment was carried out in greenhouse cabins at 10 °C at night and 20 °C during the day, with 12 h of supplementary lighting per day. At BBCH 15–16 (5–6 true leaves), five plants per pot were sampled for biometric assessment, comprising length and fresh and dry weight of the root system and of the aerial part; values were averaged per pot before analysis, as noted above. Dry weight was determined after drying at 65 °C for 14 days, to constant weight. The effect of the tested products on plant developmental parameters was expressed relative to the control using the formula: (Treatment Control)/Control × 100%. Biometric data were subjected to one-way analysis of variance (ANOVA) with treatment as the sole factor, using pot-level means (*n* = 4 pots per treatment per series) as the statistical unit; where the ANOVA was significant, means were separated using Tukey’s honestly significant difference (HSD) test at *p* < 0.05.

### 4.4. Field Experiments

The efficacy of the selected bioproducts was evaluated under field conditions on winter oilseed rape. Trials were conducted in the 2022/2023 and 2023/2024 years at two locations differing in agroclimatic conditions: the IOR-PIB Experimental Station in Winna Góra (Wielkopolskie voivodeship) and the IOR-PIB Experimental Station in Rzeszów (Skołoszów, Podkarpackie voivodeship). Trials followed EPPO methodologies PP 1/181(4), 1/78(4), 1/80(4) and 1/135(4). Winter oilseed rape cv. Artemis was grown in a randomised complete block design with four blocks per treatment at each location and season; plot size was 20 m^2^ (2.0 m × 10 m). The bioproducts and the tested product were applied twice—at BBCH 14–16 (4–6 true leaves, autumn) and at BBCH 39 (start of regrowth, spring) (Table 13). Bacterial supernatants were applied at 1% (*v*/*v*) in a spray volume of 200 L/ha, i.e., 2 L of supernatant per hectare; plant extracts were applied at 10% (*v*/*v*) in the same spray volume, i.e., 20 L of extract per hectare, following the same working solution logic used for the reference fungicide (Table 13). Treatments were applied using a Wachowiak knapsack sprayer fitted with a compressed air actuator and a spray boom with four nozzles (TeeJet DG 110-02 V). Spray volume was set at 200 L/ha, working pressure at 2.5 bar, and boom height at 50 cm above the crop canopy.

Three to four weeks after each treatment (autumn and spring), the mean percentage of leaf area affected by *Alternaria* spp. (Alternaria black spot) and by *Phoma lingam* (Phoma stem canker) was assessed, and control efficacy was calculated using Abbott’s formula (%).

Rapeseed was harvested with a Wintersteiger Classic combine harvester. Seed weight from each plot was recorded and seed moisture measured; yield was converted to t/ha at a standard seed moisture content of 11%. Random 0.5-litre seed samples were drawn from each plot; three sub-samples of 200 seeds were counted from each sample and weighed to the nearest 0.01 g on a Sartorius laboratory scale, and results were expressed as the mean weight of 1000 seeds (thousand-seed weight, TSW) in grams. Results were analysed as described above (Section 4.5).

### 4.5. Statistical Analysis

All statistical analyses for the field experiments were performed using Genstat v.24.2 statistical software package [62]. Data were primarily analysed separately for each location (Winna Góra, Rzeszów) and growing season (2022/2023, 2023/2024), following standard practice for site- and season-specific field efficacy reporting. Disease efficacy was calculated according to Abbott’s formula [63]: Efficacy (%) = [(C − T)/C] × 100, where C is the mean disease severity in the untreated control and T is the mean disease severity in the treated plots; disease severity comparisons among treatments (Table 5, Table 6 and Table 7) were conducted on plot-level severity data, and Abbott efficacy was subsequently derived from the resulting treatment means for presentation—Abbott efficacy itself is a derived descriptive statistic and does not constitute an independent replicated dataset. Disease severity data (% leaf area affected) and yield parameters (seed yield, thousand-seed weight) were subjected to one-way analysis of variance (ANOVA) within each location–season combination to evaluate the effect of treatment. Prior to ANOVA, the normality of residuals was assessed using the Shapiro–Wilk test, and homogeneity of variances was verified using Levene’s test. Where ANOVA revealed significant differences (*p* < 0.05), Tukey’s Honestly Significant Difference (HSD) post hoc test was applied for multiple pairwise comparisons between treatments. The trials were laid out as a randomised complete block design with four replicate blocks per treatment, each block consisting of a 20 m^2^ plot; in the one-way, within-location season models, treatment was fitted as the sole factor and the block term was not retained, which we acknowledge as a simplification of the underlying design. To address this, and to formally test for Treatment × Location and Treatment × Season interactions, the data were additionally analysed using combined three-way factorial ANOVA models (Type II sums of squares; effect sizes reported as eta-squared, η^2^, the proportion of total variance explained), fitted across both locations and both seasons for seed yield and thousand-seed weight (Treatment × Location × Season; Table 8) and for *Alternaria* black spot severity (Treatment × Location × Season; Table 9). Because *Phoma* stem canker was recorded only at Winna Góra, the corresponding model was Treatment × Season × Observation time (autumn/spring) (Table 10). In all combined models, the block term was retained as a fixed effect nested within environment—Block (Location × Season), df = 12, for the seed yield, TSW and *Alternaria* models, and Block (Season), df = 6, for the *Phoma* model—so that the randomised complete block structure of the trials was represented in the combined analysis (*N* = 96 in each model). This combined analysis complements, rather than replaces, the within-location–season one-way ANOVAs used for the pairwise treatment comparisons and letter groupings reported in Table 5, Table 6 and Table 7.

## 5. Conclusions

Under field conditions, the four tested bioproducts—garlic extract, Jerusalem artichoke extract, and the *Enterobacter* sp. and *Paenibacillus* sp. fermentation supernatants—provided measurable, statistically significant reductions in Phoma stem canker and Alternaria black spot severity in winter oilseed rape, in several site-years reaching a level comparable to the fungicide standard containing a.i. prothioconazole. A three-way factorial ANOVA confirmed that, in addition to a highly significant overall Treatment effect, Treatment × Location/Season and Treatment × Season/Observation-time interactions were significant for both diseases, formally supporting the qualitative observation that relative treatment performance varied across environments and assessment dates rather than remaining constant. Garlic extract and *Paenibacillus* sp. were the most consistent performers and showed the best agreement between in vitro and field activity, whereas Jerusalem artichoke extract, despite strong in vitro inhibition of *P. lingam*, translated less reliably to the field, and Enterobacter sp. remained the most pathogen- and site-dependent of the four.

The translation of disease control into yield was strongly site-dependent. At Rzeszów, the bioproducts significantly increased seed yield over the untreated control in both seasons, while at Winna Góra no yield benefit was detected, and thousand-seed weight was unaffected by treatment throughout. The three-way ANOVA confirmed that Location and Season were the dominant sources of variation for both yield endpoints (for seed yield, the Treatment effect, although significant at *p* = 0.001, explained only 3.3% of the variance; for thousand-seed weight, no Treatment effect was detected), with a significant Location × Season interaction. Overall, these results indicate that garlic extract and *Paenibacillus* sp. in particular are promising components of integrated protection programmes for winter oilseed rape, but that their yield benefit is conditional on environment—an interpretation formally supported for disease severity by the significant treatment-by-environment interactions reported above, whereas for seed yield the Treatment × Location interaction did not reach significance (*p* = 0.083) and environmental main effects dominated; multi-year, multi-site confirmation is needed before firm agronomic recommendations can be made.

## Figures and Tables

**Table 1:** Composition of the biological preparations used in the present study, summarised from [17].

Preparation	Parameter	Value
Garlic (*Allium sativum*) extract	Total phenolic content	507.2 ± 12.4 mg GAE/100 g
	Allicin	83.29 ± 0.34 mg/L
	Betulin (dominant triterpene)	275.10 ± 0.03 mg/L
	L-Aspartic acid (dominant free amino acid)	230 ± 3.5 mg/L
	D-α-Tocotrienol/D-γ-tocopherol	28.0/24.3 mg/L
	Abscisic acid	6.45 ± 2.12 µg/mL
	K/P/S/Ca/Mg	2831/860/482/348/128 mg/L
	Hg, Cr, Ni, Pb, Co	below detection limit
	Qualitative screening	saponins (+), flavonoids (+)
Jerusalem artichoke (*Helianthus tuberosus*) extract	Total phenolic content	595.7 ± 15.4 mg GAE/100 g
	Qualitative screening	saponins (+), tannins (+), flavonoids (+)
*Enterobacter* sp. supernatant (GAP1)	Glycerol	6.29 g/L
	2,3-Butanediol	2.55 g/L
	Acetic acid	1.19 g/L
*Paenibacillus* sp. supernatant (372)	Glycerol	7.85 g/L
	2,3-Butanediol	2.85 g/L
	Lactic acid	1.35 g/L
	Ethanol	1.01 g/L
	Succinic acid	0.14 g/L

**Table 2:** Effect of the tested bioproducts at three concentrations and the reference fungicide prothioconazole on inhibition of pathogen mycelial growth (%).

Pathogen	Conc. (%)	Garlic	Jerusalem Artichoke	*Enterobacter* sp.	*Paenibacillus* sp.	Prothioconazole	F	*p*-Value
*Alternaria alternata*	1	12.8 ± 2.3 d	44.8 ± 15.1 bc	31.5 ± 17.8 cd	63.0 ± 12.7 b	100.0 ± 0.0 a	46.78	<0.0001
	5	25.4 ± 5.3 c	51.3 ± 24.6 b	49.4 ± 8.1 b	48.0 ± 12.5 b	100.0 ± 0.0 a	26.29	<0.0001
	10	37.2 ± 8.5 bc	48.0 ± 11.3 b	26.5 ± 13.8 c	48.1 ± 16.4 b	100.0 ± 0.0 a	36.32	<0.0001
*Alternaria brassicicola*	1	2.6 ± 5.3 d	64.1 ± 21.3 bc	53.5 ± 24.7 c	87.6 ± 2.8 ab	100.0 ± 0.0 a	38.95	<0.0001
	5	15.2 ± 8.9 c	75.0 ± 15.3 ab	44.6 ± 40.1 bc	81.7 ± 3.4 a	100.0 ± 0.0 a	17.42	<0.0001
	10	28.5 ± 6.4 b	80.7 ± 13.4 a	38.1 ± 33.3 b	85.2 ± 3.7 a	100.0 ± 0.0 a	21.94	<0.0001
*Botrytis cinerea*	1	54.1 ± 50.5 bc	41.3 ± 11.6 c	0.0 ± 0.0 d	89.1 ± 5.0 ab	100.0 ± 0.0 a	17.66	<0.0001
	5	72.8 ± 31.6 bc	48.0 ± 13.6 c	0.0 ± 0.0 d	97.6 ± 3.7 ab	100.0 ± 0.0 a	42.95	<0.0001
	10	100.0 ± 0.0 a	55.8 ± 9.0 b	0.0 ± 0.0 c	100.0 ± 0.0 a	100.0 ± 0.0 a	860.47	<0.0001
*Phoma lingam*	1	57.8 ± 15.5 d	88.3 ± 5.0 ab	72.8 ± 3.2 c	83.0 ± 2.1 bc	100.0 ± 0.0 a	27.55	<0.0001
	5	48.1 ± 1.7 c	92.2 ± 8.8 a	70.2 ± 13.2 b	76.1 ± 2.6 b	100.0 ± 0.0 a	46.92	<0.0001
	10	50.9 ± 2.6 b	88.1 ± 13.2 a	56.1 ± 22.7 b	83.1 ± 3.3 a	100.0 ± 0.0 a	19.07	<0.0001
*Sclerotinia sclerotiorum*	1	55.6 ± 36.6 b	13.1 ± 14.7 cd	0.0 ± 0.0 d	43.0 ± 16.8 bc	100.0 ± 0.0 a	25.06	<0.0001
	5	96.9 ± 7.7 a	20.6 ± 23.3 c	0.0 ± 0.0 c	54.3 ± 24.2 b	100.0 ± 0.0 a	50.32	<0.0001
	10	100.0 ± 0.0 a	18.9 ± 16.3 c	6.3 ± 6.9 c	82.8 ± 6.0 b	100.0 ± 0.0 a	177.52	<0.0001

Different letters within a row indicate significant differences among the five treatments (Tukey HSD, *p* < 0.05). Values followed by the same letter are not significantly different (Tukey’s HSD, *p* > 0.05).

**Table 3:** Results of the greenhouse experiment—plant growth parameters (mean ± SD).

Treatment	Concentration (%)	Root Length (cm)	Shoot Length (cm)	Fresh Shoot Weight (g)	Fresh Root Weight (g)	Dry Shoot Weight (g)	Dry Root Weight (g)
Untreated control	–	12.47 ± 1.62 a	22.48 ± 4.47 b	27.34 ± 6.13 b	2.44 ± 1.00 a	2.72 ± 0.53 b	0.48 ± 0.08 a
Garlic	10	12.73 ± 2.43 a	37.64 ± 5.10 a	79.74 ± 28.96 a	3.90 ± 1.28 a	7.18 ± 2.93 a	0.70 ± 0.21 a
Jerusalem artichoke	10	14.37 ± 1.11 a	37.80 ± 3.47 a	74.70 ± 7.79 a	4.32 ± 1.14 a	6.52 ± 0.71 a	0.64 ± 0.13 a
*Enterobacter* sp.	1	13.70 ± 1.59 a	35.04 ± 6.13 a	56.96 ± 9.68 a	3.16 ± 0.67 a	5.16 ± 1.34 a	0.52 ± 0.11 a
*Paenibacillus* sp.	1	12.93 ± 1.11 a	38.20 ± 3.55 a	66.18 ± 9.42 a	3.48 ± 0.62 a	5.88 ± 1.04 a	0.56 ± 0.13 a
Prothioconazole	0.25	14.93 ± 2.02 a	33.64 ± 4.29 a	65.26 ± 7.87 a	3.68 ± 0.61 a	6.10 ± 0.76 a	0.62 ± 0.04 a

Values marked with different letters within a column differ significantly (Tukey’s HSD test, *p* < 0.05).

**Table 4:** Summary of meteorological conditions at the two experimental sites during the autumn (August–November) and spring (February–April) periods of the two growing seasons.

Location	Season	Autumn Period (August–November)		Spring Period (February–April)	
		Mean Temp. (°C)	Total Precip. (mm)	Mean Temp. (°C)	Total Precip. (mm)
Winna Góra	2022/2023	12.9	139.0	5.1	35.5
	2023/2024	13.5	149.8	8.7	130.9
Rzeszów	2022/2023	12.0	84.2	4.1	87.3
	2023/2024	11.1	182.4	8.1	146.0

**Table 5:** Efficacy of the tested treatments against Phoma stem canker (*Phoma lingam*) and Alternaria black spot (*Alternaria* spp.) at Winna Góra (mean disease severity, % leaf area; Abbott efficacy, %).

Treatment	Dose	2022/2023								2023/2024							
		Phoma (Aut.) [%]		Phoma (Spr.) [%]		Alt. (Aut.) [%]		Alt. (Spr.) [%]		Phoma (Aut.) [%]		Phoma (Spr.) [%]		Alt. (Aut.) [%]		Alt. (Spr.) [%]	
		Sev.	Eff.	Sev.	Eff.	Sev.	Eff.	Sev.	Eff.	Sev.	Eff.	Sev.	Eff.	Sev.	Eff.	Sev.	Eff.
Untreated control	—	15.00 a	—	8.00 a	—	12.00 a	—	6.00 a	—	18.00 a	—	10.00 a	—	14.00 a	—	8.00 a	—
Garlic	10%	2.40 cd	84	5.28 b	34	4.20 bc	65	4.74 b	21	3.96 c	78	5.90 bc	41	4.90 c	65	5.44 ab	32
Jerusalem artichoke	10%	6.60 b	56	6.16 ab	23	5.16 b	57	5.16 ab	14	9.72 b	46	7.20 ab	28	4.62 c	67	5.60 ab	30
*Enterobacter* sp.	1%	7.35 b	51	6.32 ab	21	8.52 a	29	5.22 ab	13	9.36 b	48	4.70 bc	53	7.14 b	49	5.36 ab	33
*Paenibacillus* sp.	1%	3.75 c	75	5.60 ab	30	2.64 c	78	4.86 ab	19	4.50 c	75	5.20 bc	48	3.08 cd	78	5.76 ab	28
Prothioconazole	0.7 L/ha	1.65 d	89	5.12 b	36	2.16 c	82	4.62 b	23	3.60 c	80	3.40 c	66	2.10 d	85	1.60 c	80

Phoma = Phoma stem canker (*Leptosphaeria* maculans); Alt. = Alternaria black spot (*Alternaria* spp.); aut. = autumn; spr. = spring. Different letters within columns indicate significant differences (Tukey HSD, *p* < 0.05). Values marked with different letters within a column differ significantly (Tukey’s HSD test, *p* < 0.05).

**Table 6:** Efficacy of the tested treatments against Alternaria black spot at Rzeszów (mean disease severity, % leaf area; Abbott efficacy, %).

Treatment	Dose	2022/2023				2023/2024			
		Autumn		Spring		Autumn		Spring	
		Sev. [%]	Eff. [%]	Sev. [%]	Eff. [%]	Sev. [%]	Eff. [%]	Sev. [%]	Eff. [%]
Untreated control	—	20.00 a	—	15.00 a	—	22.00 a	—	12.00 a	—
Garlic	10%	12.20 b	39	8.70 bc	42	9.90 b	55	6.96 ab	42
Jerusalem artichoke	10%	15.00 ab	25	2.85 d	81	9.24 b	58	7.20 ab	40
Enterobacter sp.	1%	10.60 b	47	3.75 d	75	10.56 b	52	4.56 bc	62
Paenibacillus sp.	1%	15.00 ab	25	4.05 cd	73	7.04 bc	68	6.96 ab	42
Prothioconazole	0.7 L/ha	12.20 b	39	8.70 bc	42	1.10 d	95	4.80 bc	60

Note: Phoma stem canker was not observed at this location. Different letters within columns indicate significant differences (Tukey HSD, *p* < 0.05).

**Table 7:** Seed yield (t/ha) and thousand-seed weight (TSW, g) of winter oilseed rape cv. Artemis.

Treatment	Dose	Winna Góra				Rzeszów			
		2022/2023		2023/2024		2022/2023		2023/2024	
		Yield	TSW	Yield	TSW	Yield	TSW	Yield	TSW
Untreated control	–	4.16 ab	4.77 a	2.55 a	4.91 a	4.36 c	4.46 a	3.32 c	4.22 a
Garlic	10%	3.94 b	4.82 a	2.61 a	5.01 a	4.74 ab	4.42 a	3.76 b	4.32 a
Jerusalem artichoke	10%	4.21 ab	4.99 a	2.59 a	5.08 a	5.01 ab	4.31 a	3.69 b	4.28 a
*Enterobacter* sp.	1%	3.93 b	4.64 a	2.76 a	5.19 a	4.73 b	4.34 a	3.54 bc	4.38 a
*Paenibacillus* sp.	1%	3.98 ab	4.82 a	2.55 a	5.00 a	4.78 ab	4.37 a	3.70 b	4.31 a
Prothioconazole	0.7 L/ha	4.27 a	4.84 a	2.80 a	5.10 a	5.07 a	4.33 a	4.29 a	4.36 a

Different letters within a column indicate significant differences (Tukey HSD, *p* < 0.05).

**Table 8:** Three-way ANOVA for seed yield and thousand-seed weight (TSW). Block nested within Location × Season (4 blocks per environment, df = 12). Type II SS; *N* = 96. *** *p* < 0.001, ** *p* < 0.01, * *p* < 0.05.

Source of Variation	df	Seed Yield (t/ha)			TSW (g)		
		*F*-Statistic	*p*-Value	η^2^ (%)	*F*-Statistic	*p*-Value	η^2^ (%)
Treatment (T)	5	4.74	0.001 **	3.3	0.14	0.981	0.4
Location (L)	1	182.67	<0.001 ***	25.1	65.44	<0.001 ***	35.7
Season (S)	1	305.16	<0.001 ***	41.9	6.06	0.017 *	3.3
Block (L × S)	12	9.64	<0.001 ***	15.9	2.87	0.003 **	18.8
T × L	5	2.06	0.083	1.4	0.35	0.878	1.0
T × S	5	0.67	0.645	0.5	0.71	0.622	1.9
L × S	1	24.15	<0.001 ***	3.3	10.05	0.002 **	5.5
T × L × S	5	0.61	0.695	0.4	0.23	0.950	0.6
Residual	60	–	–	8.2	–	–	32.8

**Table 9:** Three-way ANOVA for *Alternaria* black spot severity (%). Block nested within Location × Season (4 blocks per environment, df = 12). Type II SS; *N* = 96. *** *p* < 0.001, ** *p* < 0.01.

Source of Variation	df	SS	MS	*F*-Statistic	*p*-Value	η^2^ (%)
Treatment (T)	5	770.40	154.08	190.00	<0.001 ***	56.6
Location (L)	1	221.17	221.17	272.74	<0.001 ***	16.3
Season (S)	1	30.69	30.69	37.84	<0.001 ***	2.3
Block (L × S)	12	102.16	8.51	10.50	<0.001 ***	7.5
T × L	5	85.91	17.18	21.19	<0.001 ***	6.3
T × S	5	66.96	13.39	16.52	<0.001 ***	4.9
L × S	1	6.66	6.66	8.21	0.006 **	0.5
T × L × S	5	28.04	5.61	6.92	<0.001 ***	2.1
Residual	60	48.66	0.81	–	–	3.6

**Table 10:** Three-way ANOVA for *Phoma* stem canker severity (%) at Winna Góra (absent at Rzeszów). Block nested within Season (4 blocks per season, df = 6). Type II SS; *N* = 96. *** *p* < 0.001, * *p* < 0.05.

Source of Variation	df	SS	MS	*F*-Statistic	*p*-Value	η^2^ (%)
Treatment (T)	5	909.89	181.98	119.14	<0.001 ***	65.4
Season (S)	1	7.32	7.32	4.79	0.032 *	0.5
Observation time (O)	1	28.21	28.21	18.47	<0.001 ***	2.0
Block (S)	6	11.49	1.91	1.25	0.291	0.8
T × S	5	22.09	4.42	2.89	0.020 *	1.6
T × O	5	278.28	55.66	36.44	<0.001 ***	20.0
S × O	1	25.92	25.92	16.97	<0.001 ***	1.9
T × S × O	5	8.28	1.66	1.08	0.377	0.6
Residual	66	100.81	1.53	–	–	7.2

**Table 11:** Characteristics of the pathogens used in the in vitro experiment.

No.	Pathogen	Isolate Number	Origin of Isolate
1	*Alternaria alternata*	15R/2020	Rapeseed leaf
2	*Alternaria brassicicola*	2R/2020	Rapeseed leaf
3	*Botrytis cinerea*	4R/2020	Rapeseed stem
4	*Phoma lingam*	13R/2021	Rapeseed leaf
5	*Sclerotinia sclerotiorum*	11R/2021	Rapeseed stem

**Table 12:** Characteristics of the natural products used in vitro experiments and greenhouse experiments.

No.	Product Name	Concentration (After Dilution of Initial Extract) % (*v*/*v*)	Selected Concentration in Greenhouse Studies % (*v*/*v*)	Additional Data
1	Garlic (*A. sativum*) extract	1, 5, 10	10	Water extract of garlic (bulb), DER 1:2.5; ultrasound-assisted extraction; conditions: extraction time: 1 h, temperature: 60 °C
2	Jerusalem artichoke (*H. tuberosus*) extract	1, 5, 10	10	Water extract of Jerusalem artichoke (leaves), DER 1:9; ultrasound-assisted extraction; conditions: extraction time: 1 h, temperature: 60 °C
3	*Enterobacter* sp. supernatant	1, 5, 10	1	Fermentation broth (GAP1) obtained by *Enterobacter* bacteria in a bioreactor
4	*Paenibacillus* sp. supernatant	1, 5, 10	1	fermentation broth (372) obtained by the *Paenibacillus* bacteria in a bioreactor
5	Prothioconazole	0.25	0.25	Protikon 250 EC (250 g active ingredient/L)

**Table 13:** Field experiment design.

No.	Test Object	Concentration/Rate Per ha	Application Timing	
			Autumn BBCH 14-16 (Autumn)	Regrowth BBCH 39 (Spring)
1	Control (untreated)	–	–	–
2	Garlic (*A. sativum*) extract	10%	+	+
3	Jerusalem artichoke (*H. tuberosus*) extract	10%	+	+
4	*Enterobacter* sp. supernatant	1%	+	+
5	*Paenibacillus* sp. supernatant	1%	+	+
6	Prothioconazole (250 g/L)—(Protikon 250 EC)	0.7 L/ha	+	+

## Data Availability

The data presented in this study are available on request from the corresponding author. The chemical characterisation data summarised in Table 1 are reported in full in the previously published companion study [17].

## Abbreviations

The following abbreviations are used in this manuscript:

TSW	Thousand-seed weight
TPC	Total phenolic content
HSD	Tukey’s honestly significant difference test
DER	Drug-to-extract ratio
